# Supplementary material for: Impact of Viral Co-Detection on the Within-Host Viral Diversity of Influenza Patients
Source: Viruses. 2025 Jan 23;17(2):152. doi: 10.3390/v17020152 (PMC11861327; doi:10.3390/v17020152)
Supplement: Supplementary file 1 [file viruses-17-00152-s001.zip › viruses-3337799-supplementary.pdf]

Supplementary File

Impact of viral co-detection on within-host viral diversity of influenza patients

Su Myat Han [1,2,*], Yoshiano Kubo [3,4], Alexis Robert [2,5], Marc Baguelin [2,6] and Koya Ariyoshi [1,4,5]

[1] School of Tropical Medicine and Global Health, Nagasaki University, Nagasaki, 852-8523 Japan: koya.ariyoshi@gmail.com (K.A.) pearl.june@gmail.com (SMH)

[2] Department of Infectious Disease Epidemiology, Faculty of Epidemiology and Population Health, London School of Hygiene and Tropical Medicine, London, WC1E 7HT UK; marc.baguelin@imperial.ac.uk Alexis.Robert@lsthm.ac.uk (A.R) pearl.june@gmail.com (SMH)

[3] Graduate School of Biomedical Sciences, Nagasaki University, Nagasaki, 852-8520 Japan; yoshinao@nagasaki-u.ac.jp

[4] Department of Clinical Medicine, Institute of Tropical Medicine, Nagasaki University, Nagasaki, 852-8102 Japan; koya.ariyoshi@gmail.com (K.A.) yoshinao@nagasaki-u.ac.jp

[5] Infectious Disease Epidemiology and Dynamics, Institute of Tropical Medicine, Nagasaki University, Nagasaki, 852-8102 Japan; koya.ariyoshi@gmail.com (K.A.) Alexis.Robert@lshtm.ac.uk (A.R.)

[6]MRC Centre for Global Infectious Disease Analysis; and the Abdul Latif Jameel Institute for Disease, Imperial College London, UK SW7 2AZ, UK marc.baguelin@imperial.ac.uk

* Correspondence: pearl.june@gmail.com

Supplementary Table S1: Multiplex results of the respiratory pathogens (2012-13 season)

Age group	pop	ILI	IAV	IBV	PIV	RV	Adeno	Boca virus	hMPV	hCoV	hRSV	No pathogen detected
<5	479	444	62	5	20	**77**	15	7	2	5	5	271
5-10	686	328	**108**	4	3	42	10	5	3	1	2	163
11-15	845	289	**70**	4	5	35	2	6	1	0	2	176
16-20	607	164	**44**	3	2	18	2	3	0	0	4	100
21-25	114	40	9	0	0	6	2	0	0	0	1	27
26-30	227	62	18	0	0	7	0	0	0	0	0	39
31-35	329	100	28	4	1	7	1	0	0	0	0	63
36-40	500	101	33	1	1	9	1	0	0	1	2	56
41-45	555	90	30	2	1	9	0	0	0	2	0	50
46-50	615	85	36	1	3	9	3	2	0	1	0	36
51-55	675	60	23	1	0	2	1	1	0	0	1	34
56-60	770	59	21	1	1	8	0	0	0	0	0	33
61-65	920	46	12	1	1	2	0	0	0	0	0	30
66-70	869	53	12	1	1	6	1	0	0	0	0	34
71-75	1091	54	9	1	0	2	0	0	0	0	0	43
>75	3340	338	31	1	3	25	6	2	2	4	3	270

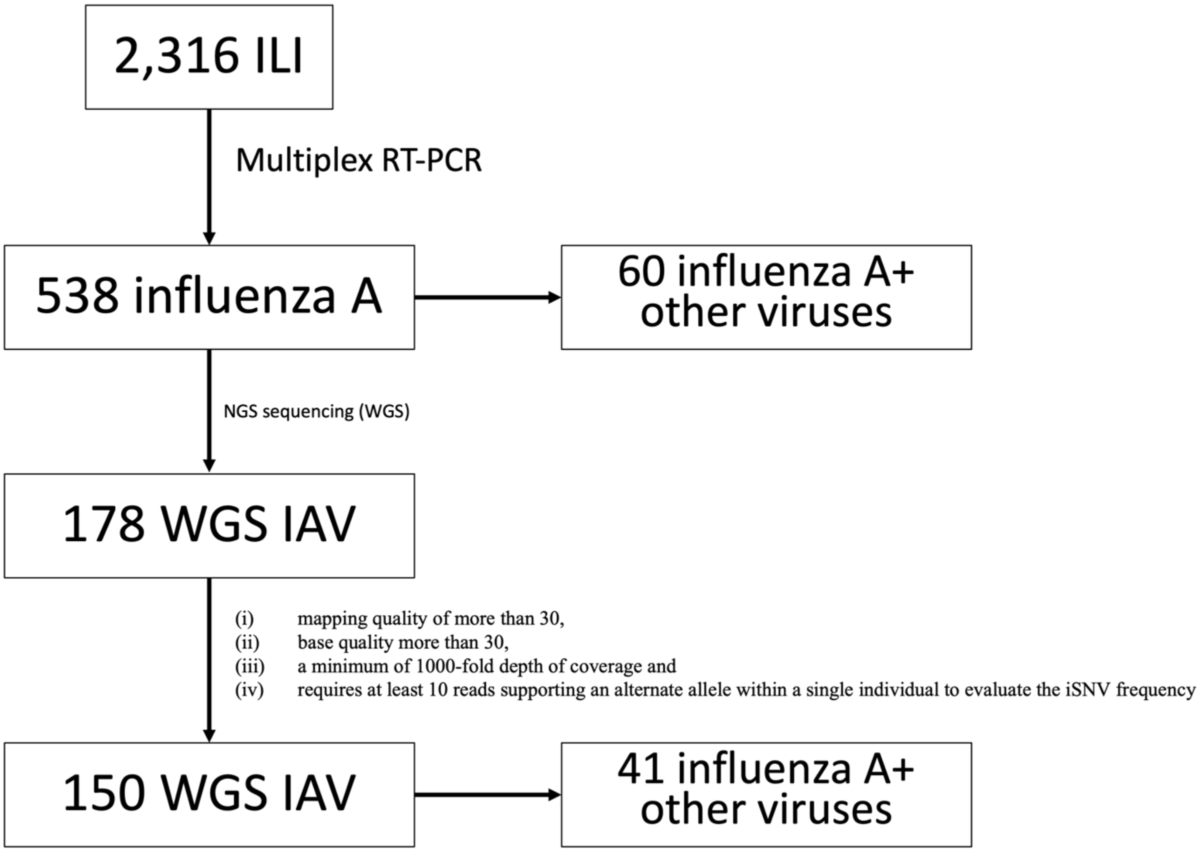

Supplementary Figure S1: Flowchart showing the final results of WGS samples available for the downstream analysis in the study

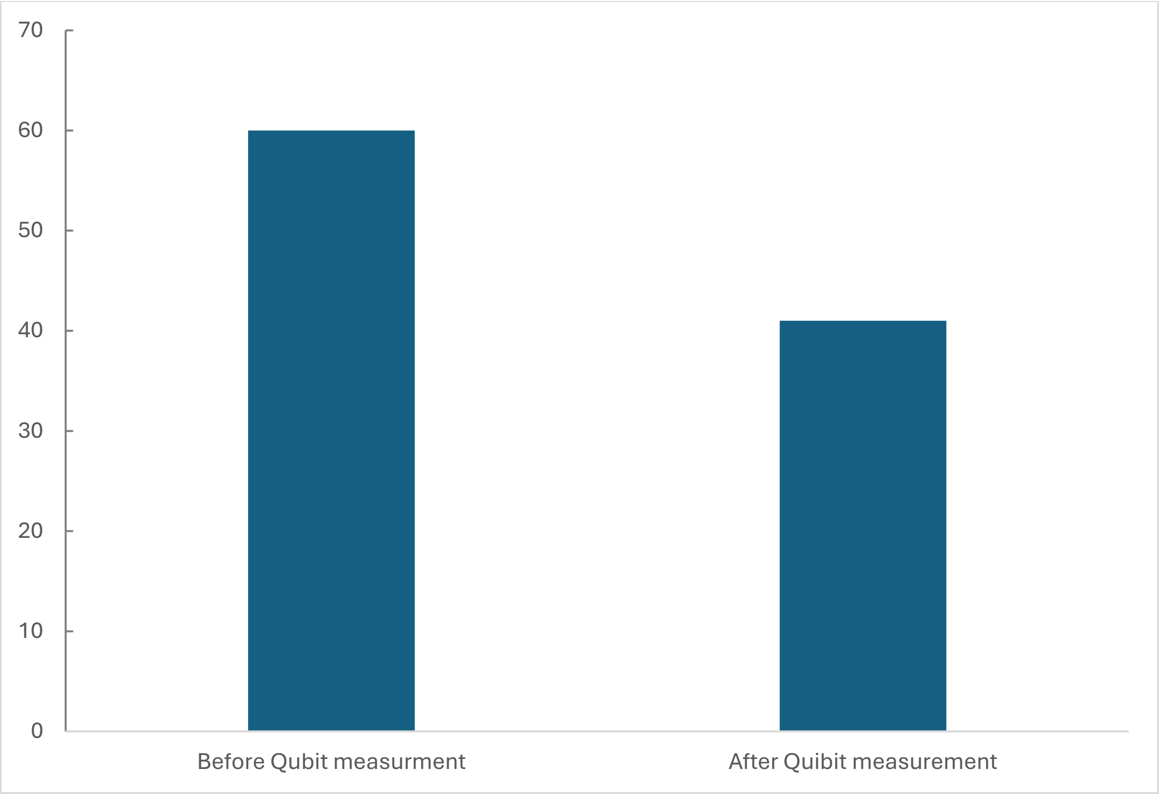

Supplementary Figure S2: Number of codetection samples available for WGS (before and after measuring with Qubit for ng/ul)
**samples with at least 1ng/ul are used for further WGS

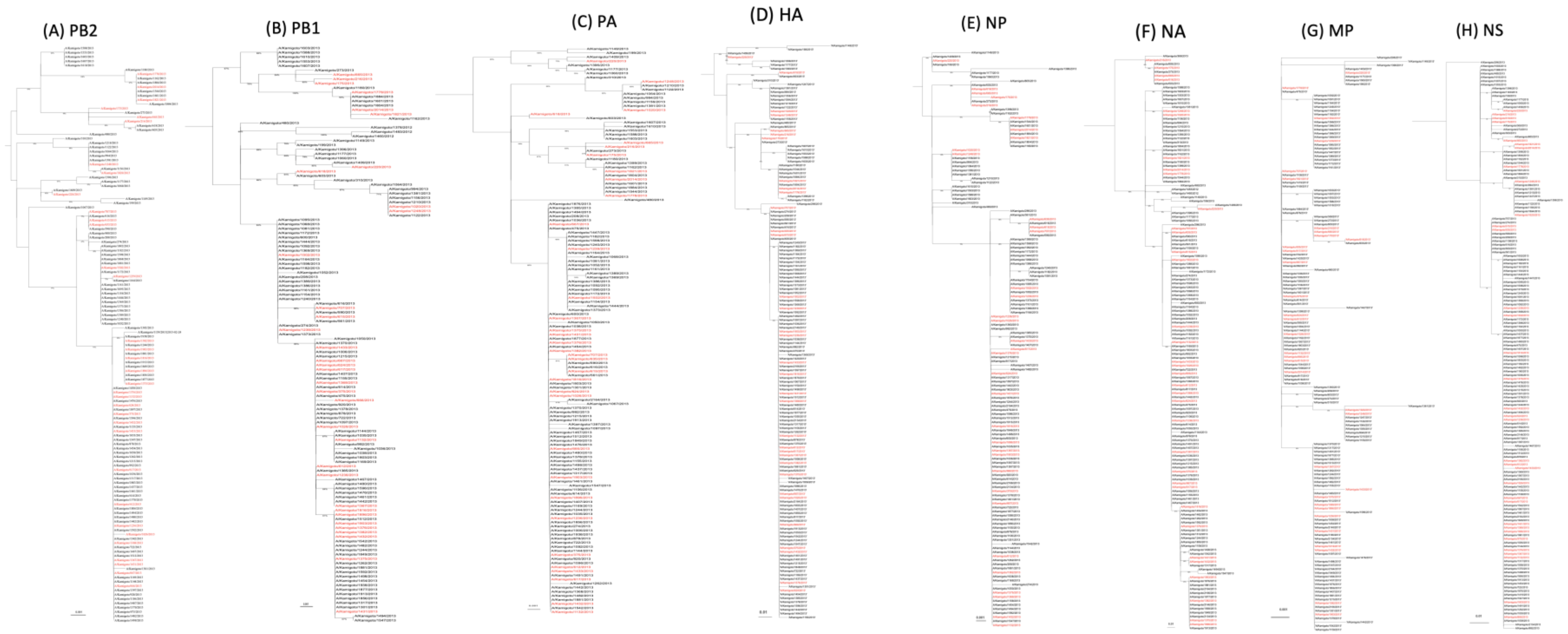

Supplementary Figure S3: Maximum likelihood phylogenetic tree of (A) PB2, (B) PB1, (C) PA, (D) HA, (E) NP, (F) NA, (G) MP, (H) NS segment of A/H3N2 sequences in 2012/13 influenza season. The sequences in red color are virus-virus co-detected and black color are only influenza.

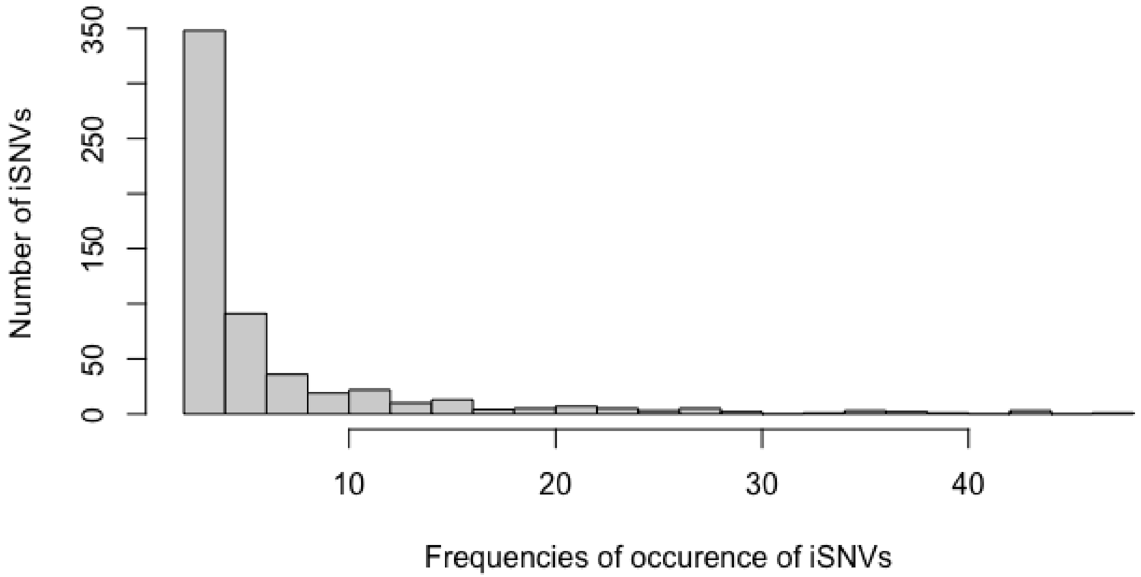

Supplementary Figure S4: Number of occurrences of iSNV
